# Recovery and Purification of Lithium Hydroxide from Spent Cathode Crucibles via Sulfation and Conversion Processes

**DOI:** 10.3390/ma19112252

**Published:** 2026-05-26

**Authors:** Jin-Seong Yoon, H. Y. Sohn, Jei-Pil Wang

**Affiliations:** 1 Department of Metallurgical Engineering, BB21 Plus Team, Pukyong National University, Busan 48513, Republic of Korea; 2 Departments of Materials Science and Engineering and of Chemical Engineering, University of Utah, Salt Lake City, UT 84112, USA

**Keywords:** lithium hydroxide, lithium sulfate, spent cathode crucibles, sulfuric acid leaching, ion exchange, impurity removal, double-displacement reaction, barium hydroxide

## Abstract

This study presents an integrated process for the recovery and purification of lithium hydroxide (LiOH) from lithium sulfate (Li_2_SO_4_) solution obtained by sulfuric acid leaching of spent crucibles used for producing the cathodes of LIBs. The recovered leachate contains considerable concentrations of metallic impurities, including Na, K, Mg, Ca, Al, and Ni, which hinder the direct production of high-purity LiOH. To overcome this limitation, a pretreatment step combining cation- and anion-exchange resins was introduced to control impurity levels and condition the solution prior to conversion. Under the optimized ion-exchange condition of 10 g cation-exchange resin and 50 g anion-exchange resin, the solution pH was adjusted to 6–7, resulting in effective impurity removal through combined ion-exchange and solution-conditioning effects. More than 90% of Al was removed, while Mg, Ca, Na, K, and Ni were removed by approximately 70–75%. After purification, LiOH was produced through a double-displacement conversion reaction using Ba(OH)_2_. The results showed that the reaction temperature and the [OH]:[Li] molar ratio were the key parameters governing the sulfate-removal-based apparent conversion efficiency and filtrate-based LiOH purity. Excess OH^−^ promoted the formation of dissolved and complexed species, thereby lowering the purity of the LiOH-containing filtrate. In contrast, the optimum condition was identified at 70 °C and an [OH]:[Li] molar ratio of 1:1, under which SO_4_^2−^ was effectively removed as solid BaSO_4_. Under these conditions, the sulfate-removal-based apparent conversion efficiency reached 91.91%, and the filtrate-based LiOH purity was 98.84%. X-ray diffraction analysis confirmed the coexistence of LiOH·H_2_O and LiOH phases in the final recovered product, whereas the precipitate was identified as single-phase BaSO_4_, indicating effective sulfate removal. Overall, this study demonstrates the feasibility of producing high-purity LiOH from sulfation-derived Li_2_SO_4_ leachate through a sequential process consisting of impurity removal, conversion, and drying. The findings provide fundamental process data for the design of lithium recovery and purification routes using spent cathode crucibles as secondary lithium resources.

## 1. Introduction

The accelerating global transition toward carbon neutrality and climate-change mitigation has markedly increased the demand for lithium-ion batteries (LIBs), particularly for electric vehicles (EVs) and large-scale energy storage systems (ESSs) [1]. In parallel, the industrial importance of lithium hydroxide (LiOH) has grown rapidly, and recent reviews have highlighted its increasing role as a key lithium precursor for advanced battery applications [2]. Among various cathode chemistries, Ni-rich layered cathodes have attracted considerable attention because of their high energy density and reduced cobalt dependency [3,4,5]. In particular, recent studies have shown that advanced Ni-rich cathodes can achieve improved cycling stability and high capacity, thereby further increasing the demand for high-purity lithium precursors for next-generation battery manufacturing [3,4,5]. In this context, LiOH has become increasingly important because it is more suitable than lithium carbonate (Li_2_CO_3_) for the synthesis of Ni-rich cathode materials owing to its higher reactivity and lower calcination temperature requirement [2,6].

In conventional lithium-ion battery chemistry, LiOH is not generally used as a cathode active material itself, but serves as a key lithium precursor for the synthesis of layered oxide cathode materials, particularly Ni-rich NCM and NCA cathodes. Compared with Li_2_CO_3_, LiOH can react more readily with transition-metal hydroxide or oxide precursors during calcination, enabling lower synthesis temperatures and improved lithiation efficiency. Therefore, the purity of LiOH is important because residual impurities such as Na, K, Ca, Mg, Al, Ni, sulfate species, or other soluble residues may affect precursor mixing, phase formation, calcination behavior, and the electrochemical performance of the final cathode material. For this reason, the recovery of LiOH from secondary lithium resources should be accompanied by careful impurity control and purification.

Conventional lithium supply relies mainly on brines and hard-rock ores; however, these primary resources are associated with uneven geographical distribution, energy-intensive processing, and environmental burdens [2,7,8]. At the same time, the rapid expansion of the battery industry has intensified concerns regarding the stable supply of critical minerals such as lithium, nickel, cobalt, and manganese. Consequently, growing attention has been directed toward secondary lithium resources and recycling-based recovery routes [9,10,11,12]. Recent reviews have emphasized that sustainable lithium recovery should combine high resource efficiency, product purity, and flexible process integration, especially for the production of battery-grade lithium compounds [10,11,12].

LIBs have become the dominant power source for EVs and ESSs because of their high energy density and long cycle life [1]. In particular, the market share of Ni-rich cathodes, such as NCM and NCA, has continued to grow because these materials provide higher energy density than conventional LCO, LMO, and LFP cathodes [3,4,5]. However, the synthesis of Ni-rich cathodes requires high-purity lithium precursors, and the carryover of residual lithium or impurities can adversely affect cathode structural stability and electrochemical performance [6]. As a result, the global demand for LiOH has steadily increased, and considerable efforts have been devoted to developing efficient routes for LiOH production from both primary and secondary lithium sources [2,13,14,15,16].

In cathode manufacturing plants, prolonged high-temperature calcination can cause lithium-containing compounds to accumulate on and react with aluminosilicate-based reaction vessels or sagger crucibles. Consequently, spent cathode crucibles are periodically generated as lithium-bearing industrial waste. These spent reaction vessels, generally discarded after simple crushing or landfilling, still contain approximately 1–2 wt% lithium. This indicates that spent cathode crucibles are not merely waste materials, but potentially valuable secondary lithium resources. Nevertheless, compared with end-of-life LIBs, limited attention has been paid to lithium recovery from spent reaction vessels [9,10,11,12]. Recent work has demonstrated that lithium can be recovered from spent lithium-containing sagger crucibles and converted into battery-grade LiOH·H_2_O through sulfation-based processing and double-decomposition chemistry, confirming the feasibility of this waste stream as an alternative lithium source [17].

Several approaches have been proposed for LiOH production from lithium-bearing sulfate media. A recent review categorized LiOH production routes into electrochemical conversion, precipitation-based methods, and selective extraction processes, highlighting the growing industrial importance of high-purity LiOH production [2]. Electrochemical routes, including bipolar membrane electrodialysis and related membrane-based processes, have been investigated for converting Li_2_SO_4_ into LiOH [13,14,15,16]. However, membrane selectivity, ion transport behavior, and process optimization remain critical challenges for practical implementation [13,14,15,16]. Alternatively, precipitation conversion using alkaline reagents has been reported as an effective route for producing LiOH from lithium sulfate solution [17,18]. In particular, the use of Ba(OH)_2_ enables sulfate removal through the formation of insoluble BaSO_4_, offering a relatively simple and efficient conversion pathway [17,18].

Despite these advantages, the quality of the final LiOH product strongly depends on the impurity level of the lithium sulfate feed solution. Metallic impurities such as Na, K, Mg, Ca, Al, and Ni can deteriorate precursor quality and may eventually degrade the structural stability and electrochemical performance of Ni-rich cathodes [3,4,5,6]. Therefore, impurity removal is an essential step in any practical LiOH recovery process. In this regard, ion-exchange treatment is a promising pretreatment strategy because it can selectively remove dissolved ionic impurities while minimizing lithium loss [19,20]. Based on these considerations, this study investigates the recovery and purification of lithium hydroxide from Li_2_SO_4_ leachate derived from spent cathode crucibles. The specific objectives are as follows: (i) to remove dissolved impurities from the lithium sulfate leachate using ion-exchange resins; (ii) to convert the purified Li_2_SO_4_ solution into a LiOH solution through a double-displacement reaction with Ba(OH)_2_ accompanied by BaSO_4_ precipitation; and (iii) to recover a final LiOH product after evaporation and drying. By clarifying the effects of impurity control, reaction temperature, and [OH]:[Li] molar ratio on conversion efficiency and product purity, this study aims to generate fundamental process data for the valorization of spent cathode crucibles as secondary lithium resources and for the design of practical LiOH recovery processes [17,18].

Although BaSO_4_-driven double-displacement conversion has previously been reported for LiOH·H_2_O recovery from spent lithium-containing sagger crucibles, the present study differs in its focus on the purification and conditioning of impurity-rich Li_2_SO_4_ leachate prior to LiOH production. In practical leachate systems, metallic impurities such as Al, Mg, Ca, Na, K, and Ni can significantly affect the quality of the LiOH-containing solution and the subsequent crystallized product. Therefore, the novelty of this work lies in the systematic integration of cation-exchange and anion-exchange pretreatment with Ba(OH)_2_-mediated precipitation conversion, with particular emphasis on impurity removal, lithium-loss behavior, residual sulfate control, and the limitations of filtrate-based LiOH-equivalent purity. This study therefore provides additional process-level insight into impurity control and purification requirements for converting spent-crucible-derived Li_2_SO_4_ leachate into LiOH-containing products.

## 2. Materials and Methods

### 2.1. Overview

Lithium hydroxide was produced from a lithium sulfate (Li_2_SO_4_) leachate obtained from spent cathode crucibles. The experimental procedure consisted of three sequential steps: (i) characterization of the Li_2_SO_4_ leachate, (ii) impurity removal by ion-exchange treatment, and (iii) conversion of the purified Li_2_SO_4_ solution to LiOH solution, followed by solid–liquid separation and crystallization/drying.

#### 2.1.1. Characterization of the Feed Solution

The feed solution used in this study was a Li_2_SO_4_ leachate prepared by sulfuric acid leaching of crushed spent reaction vessels. The leachate solution was produced by adding 1 kg of crushed powder to 900 mL of distilled water mixed with 100 mL of 98 wt% H_2_SO_4_. To determine the concentrations of lithium and impurity elements in the leachate, inductively coupled plasma-optical emission spectroscopy (ICP-OES, Agilent, Santa Clara, CA, USA) was employed. The sulfate ion concentration was additionally analyzed by ion chromatography (IC). Prior to analysis, the leachate was filtered through a hydrophilic membrane filter with a pore size of 0.2 μm. The chemical composition of the Li_2_SO_4_ leachate is summarized in Table 1. The major impurities identified in the feed solution were Al, Mg, Ca, Na, K, and Ni, together with a high concentration of SO_4_^2−^.

#### 2.1.2. Ion-Exchange Treatment

The ion-exchange treatment was performed to remove dissolved metallic impurities from the Li_2_SO_4_ leachate prior to LiOH conversion. A magnetic stirrer was used to ensure uniform mixing of the solution and ion-exchange resins under ambient conditions. The reaction was carried out in a 500 mL glass beaker equipped with a magnetic bar, and the stirring speed was fixed at 200 rpm.

#### 2.1.3. LiOH Conversion System

**Figure 1:**
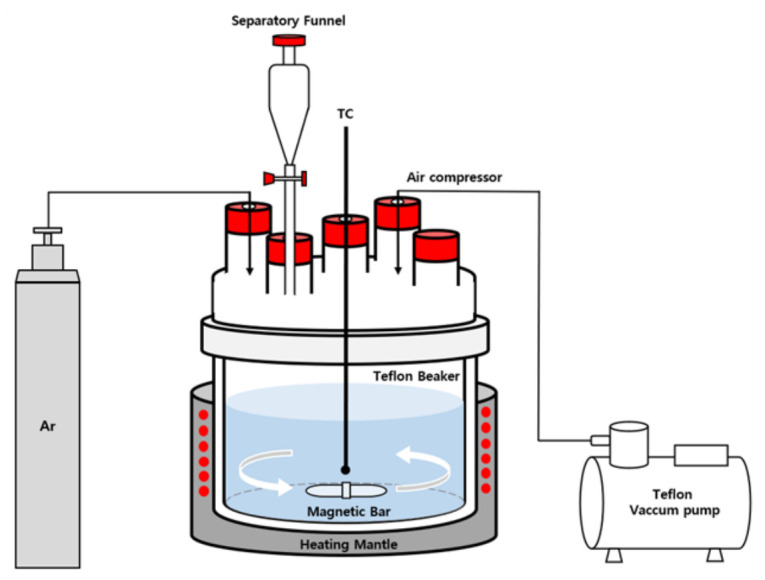
Schematic diagram of the Teflon reaction vessel system used for the conversion of Li_2_SO_4_ to LiOH.

A schematic diagram of the reaction system used for the conversion of Li_2_SO_4_ to LiOH is shown in Figure 1. The conversion reactor was designed to provide uniform mixing, temperature control, and atmospheric control during the reaction to prevent the LiOH solution from reacting with atmospheric CO_2_. A chemically inert Teflon reactor, instead of the typical glass vessel, was used because the lithium ions may react with SiO_2_ from glass to form lithium silicate. The reactor was heated using a heating mantle, and the reaction temperature was controlled in the range of 60–80 °C. A magnetic bar was placed in the Teflon beaker to ensure homogeneous mixing. The Li_2_SO_4_ solution was introduced dropwise through a separatory funnel. To minimize contamination and suppress the ingress of oxygen and moisture, the reactor was purged with Ar gas, and, when necessary, a vacuum-Ar atmosphere was established using a Teflon vacuum pump. The internal temperature was continuously monitored using a thermocouple.

#### 2.1.4. Solid–Liquid Separation and Drying

After the LiOH conversion reaction, the slurry product was separated by vacuum filtration using a Büchner funnel, a side-arm Erlenmeyer flask, and a Teflon vacuum pump. A PTFE hydrophilic membrane filter with a pore size of 0.2 μm was used to separate the BaSO_4_ precipitate from the LiOH-containing filtrate. Because LiOH solution readily reacts with atmospheric CO_2_, exposure to air was minimized during product recovery. Immediately after filtration, the recovered LiOH solution was vaporized under an Ar-vacuum atmosphere to obtain LiOH·H_2_O crystals. This procedure was adopted to suppress carbonation during crystallization and drying, thus preventing deterioration of product purity.

### 2.2. Experimental Procedure

#### 2.2.1. Process Flow for LiOH Production

**Figure 2:**
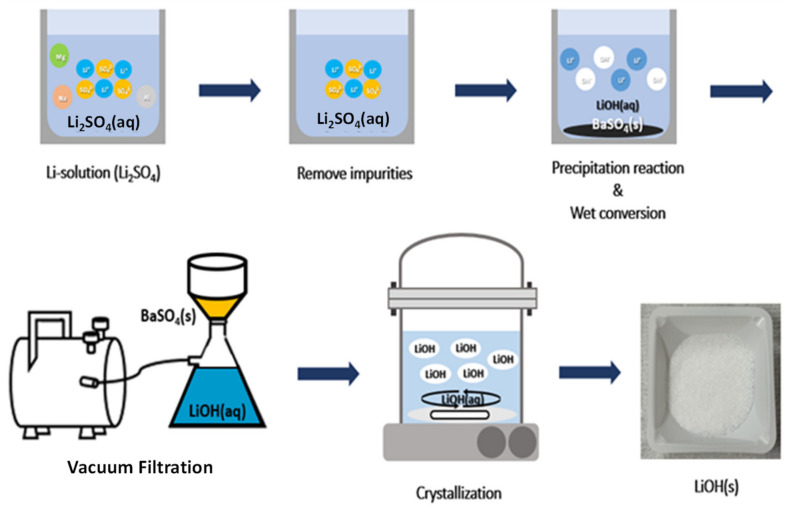
Schematic process flow for the recovery of LiOH from Li_2_SO_4_ leachate derived from spent reaction vessels through impurity removal, precipitation conversion, vacuum filtration, and crystallization.

In this study, high-purity lithium hydroxide was produced using a lithium sulfate (Li_2_SO_4_) solution obtained from spent reaction vessels through sulfuric acid leaching as the starting material. The overall process consisted of three sequential steps: (i) impurity removal, (ii) alkaline precipitation and conversion to LiOH, and (iii) vacuum evaporation and crystallization. A schematic flow diagram of the overall process is presented in Figure 2. In the impurity-removal step, three different ion-exchange treatments were carried out depending on the resin treatment condition. First, a cation-exchange resin (MC-10H) was used to remove major cationic impurities and to evaluate the primary removal effect. Subsequently, an anion-exchange resin (MA-10OH) was applied to control the SO_4_^2−^ concentration and solution pH, while also examining its effect on additional impurity removal. Finally, combined treatment using both cation- and anion-exchange resins was performed to optimize the overall impurity-removal efficiency. The purified solution was then subjected to a double-displacement reaction using barium hydroxide [Ba(OH)_2_] as an alkaline precipitating agent. During this step, sulfate ions were removed through BaSO_4_ precipitation, while the lithium sulfate solution was converted into a lithium hydroxide solution. After the reaction, the generated BaSO_4_ precipitate was separated by vacuum filtration, and the LiOH-containing filtrate was recovered. Finally, the recovered solution was evaporatively dried under vacuum and an inert atmosphere to obtain LiOH·H_2_O crystals while suppressing carbonation by atmospheric CO_2_. The compositional changes before and after each processing step were analyzed by ICP-OES, IC, and XRD, and the impurity-removal efficiency, product purity, and LiOH conversion efficiency were evaluated. Each experimental condition in the ion-exchange treatment and Ba(OH)_2_ precipitation-conversion tests was conducted as a single experimental run. Therefore, standard deviations or error bars were not calculated. The experiments were designed primarily to identify process trends and suitable operating windows for impurity removal, LiOH conversion, and purity improvement, rather than to provide statistically validated reproducibility data.

#### 2.2.2. Impurity Removal from the Li_2_SO_4_ Leachate

In this study, an ion-exchange process was applied to remove impurities from the lithium sulfate solution obtained through sulfuric acid leaching. Cation-exchange resins generally exhibit high selectivity toward multivalent cations and therefore preferentially remove ions such as Al^3+^, Mg^2+^, Ca^2+^, and Ni^2+^. In contrast, alkali metal ions such as Li^+^ show relatively low affinity for the resin, resulting in limited removal. This characteristic is advantageous for minimizing lithium loss while improving the purity of the feed solution for subsequent conversion. The ion-exchange resins used in this study were Trilite^®^ (Samyang Corp., Seoul, Republic of Korea) MC-10H (H-form cation-exchange resin) and Trilite^®^ MA-10OH (OH-form anion-exchange resin). These resins were selected by considering the composition of the sulfuric acid leachate, the valence difference in the target ions to be removed, and the solution conditions required for the subsequent Ba(OH)_2_ precipitation reaction. MC-10H is a strongly acidic cation-exchange resin containing –SO_3_H functional groups. As shown in Equation (1), it effectively removes metallic impurities through ion exchange with H^+^, particularly exhibiting high selectivity toward multivalent cations such as Al^3+^, Mg^2+^, Ca^2+^, and Ni^2+^. In contrast, Li^+^, as a monovalent cation, has relatively low affinity for the resin and therefore tends to remain in solution, making this resin suitable for pretreatment with minimal lithium loss

zR−SO_3_H + M^z+^ → (R−SO_3_)zM + zH^+^ (1)

where $M^{z+}$ denotes a metal cation with charge z+.

MA-10OH is a strongly basic anion-exchange resin containing –OH functional groups. As shown in Equation (2), this resin exchanges sulfate ions while releasing OH-, thereby increasing the pH of the initially strongly acidic solution toward the near-neutral range. Such pH adjustment plays an important role in providing a favorable reaction environment for the subsequent BaSO_4_ precipitation and LiOH conversion step.

2R−OH + SO_4_^2−^ → R2−SO_4_ + 2OH^−^ (2)

The amount of resin added was determined by considering the concentrations of the major ions in solution and the exchange capacities of the resins. The resin dosage was estimated using Equation (3), where $W_{resin}$ is the required mass of ion-exchange resin (g), f is the target removal fraction, $n_{ion}$ is the total equivalent amount of major ions present in the solution (eq), and $C_{resin}$ is the exchange capacity of the resin per unit mass (eq g^−1^). This equation was used to estimate the resin dosage required to achieve the target removal efficiency based on the total ionic equivalents in the solution and the effective exchange capacity of the resin.

(3) $$W_{resin(g)}=\frac{f\times n_{ion}(eq)}{C_{resin(\frac{eq}{g})}}$$

For 50 mL of the lithium leachate used in this study, the total equivalent concentration of the major cationic impurities (Al^3+^, Mg^2+^, Ca^2+^, Na^+^, K^+^, and Ni^2+^) was calculated to be approximately 0.0288 eq, whereas that of SO_4_^2−^ was approximately 0.104 eq. The effective exchange capacities applied in this study were 0.0015 eq g^−1^ for Trilite^®^ MC-10H and 0.000975 eq g^−1^ for Trilite^®^ MA-10OH. Based on these values, the baseline resin dosages were determined. The impurity-removal experiments were conducted in three different modes: cation-exchange treatment alone, anion-exchange treatment alone, and combined treatment using both cation- and anion-exchange resins. For the cation-only experiments, the resin dosage was varied in the range of 10–50 g. For the anion-only experiments, the dosage was varied from 30 to 70 g. Because the initial pH of the leachate was approximately 1, a somewhat higher dosage range was applied for the anion-exchange resin than the calculated theoretical value. Finally, simultaneous treatment with both cation- and anion-exchange resins was performed to determine the optimum resin combination based on the behavior observed in the single-resin experiments.

### 2.3. Thermodynamic Consideration Related to Conversion of Li_2_SO_4_ to LiOH

To convert the Li_2_SO_4_ to LiOH, the removal of sulfate ions from the solution is essential. In this study, barium hydroxide [Ba(OH)_2_] was employed as the precipitating agent for sulfate removal and conversion to LiOH. Ba(OH)_2_ is a strong base that stabilizes Li^+^–OH^−^ species in solution while selectively reacting with sulfate ions to form stable BaSO_4_ precipitates. Because BaSO_4_ has an extremely low solubility in water, it can be readily separated as a solid phase after the reaction, thereby enabling effective sulfate removal from the Li_2_SO_4_ solution. Compared with other alkaline-earth hydroxides such as Ca(OH)_2_ and Mg(OH)_2_, Ba(OH)_2_ offers several advantages for this conversion process. In particular, its sulfate reaction product, BaSO_4_, is significantly less soluble than CaSO_4_ or MgSO_4_, which facilitates more efficient sulfate removal. In addition, Ba(OH)_2_ is less likely to induce unnecessary competing reactions with metallic impurities, thereby minimizing disturbance to the Li^+^–OH^−^ equilibrium. For these reasons, Ba(OH)_2_ was considered the most suitable precipitating agent for LiOH production from Li_2_SO_4_ solution in terms of sulfate-removal efficiency, selectivity, and solution stability.

The conversion reaction is based on a double-displacement reaction between lithium sulfate and barium hydroxide, as expressed in Equation (4). In this reaction, BaSO_4_ is separated as a solid precipitate because of its very low water solubility, while LiOH remains in the aqueous phase. This step is therefore considered the key reaction for producing a high-purity LiOH solution from Li_2_SO_4_ leachate.

Li_2_SO_4_(aq) + Ba(OH)_2_ → 2LiOH(aq) + BaSO_4_(s) (4)

**Figure 3:**
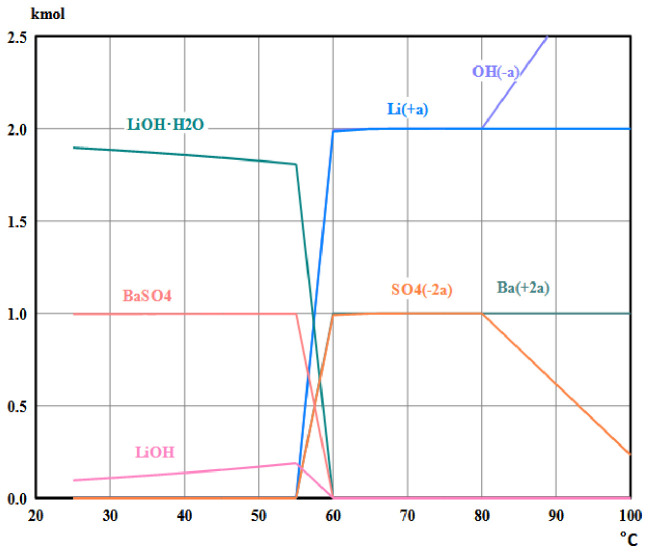
Calculated equilibrium concentration profiles for the reaction between Li_2_SO_4_ and Ba(OH)_2_ as functions of temperature.

Figure 3 shows the calculated equilibrium concentration profiles for the reaction between Li_2_SO_4_ and Ba(OH)_2_ as a function of temperature, obtained using HSC Chemistry 6. At lower temperatures, the calculated phase distribution indicates incomplete stabilization of the LiOH-related species. However, above approximately 60 °C, the concentrations of Li^+^ and OH^−^ became nearly constant, suggesting that the LiOH-forming aqueous species were thermodynamically stable in this range. Accordingly, the reaction temperature for the double-displacement conversion was selected as 60–80 °C in this study.

**Figure 4:**
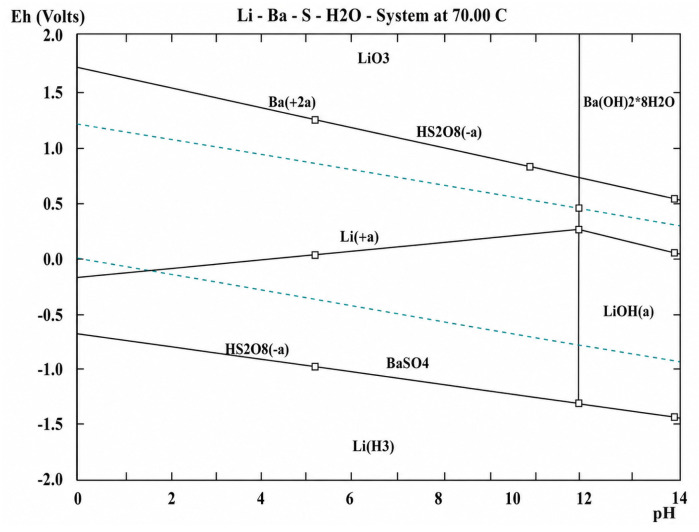
Eh–pH diagram of the Li–Ba–S–H_2_O system showing the stability region of LiOH and BaSO_4_.

In addition, the Eh–pH diagram of the Li–Ba–S–H_2_O system shown in Figure 4 was used to examine the stability region of LiOH and BaSO_4_. LiOH(aq) was predicted to be stable in the strongly alkaline region above approximately pH 12, whereas BaSO_4_ remained stable as a solid phase over a relatively wide pH range. Upon addition of Ba(OH)_2_, the solution pH was expected to shift toward the thermodynamically favorable region for LiOH stability, thereby providing appropriate conditions for the precipitation-conversion reaction. Accordingly, the conversion reaction was carried out at 60–80 °C, where the reactants were expected to be sufficiently ionized and the LiOH-forming aqueous species were thermodynamically stable. After the reaction, the BaSO_4_ precipitate and the LiOH solution were separated by vacuum filtration. The recovered filtrate was subsequently evaporatively dried under a vacuum-Ar atmosphere to obtain the final solid LiOH·H_2_O and LiOH products.

## 3. Results and Discussion

### 3.1. Impurity Removal by Ion-Exchange Resins

#### 3.1.1. Cation-Exchange Treatment with MC-10H Resin

To remove cationic impurities from the Li_2_SO_4_ solution, Trilite^®^ MC-10H cation-exchange resin was applied. The resin dosage was varied from 10 to 50 g, and all experiments were carried out using 50 mL of the Li_2_SO_4_ leachate. The initial solution was strongly acidic (pH 1), under which the metallic cation species remained dissolved. The experimental conditions are summarized in Table 2.

Table 3 summarizes the concentration changes in major ionic species after cation-exchange treatment with different MC-10H dosages. As the resin dosage increased, the concentrations of major impurities, including Al, Mg, Ca, Na, Ni, and K, decreased markedly. At the highest dosage of 50 g, the concentrations of Al, Mg, Ca, Na, Ni, and K decreased to 174.07, 125.87, 15.81, 34.20, 19.64, and 15.04 mg kg^−1^, respectively, indicating substantial removal.

**Figure 5:**
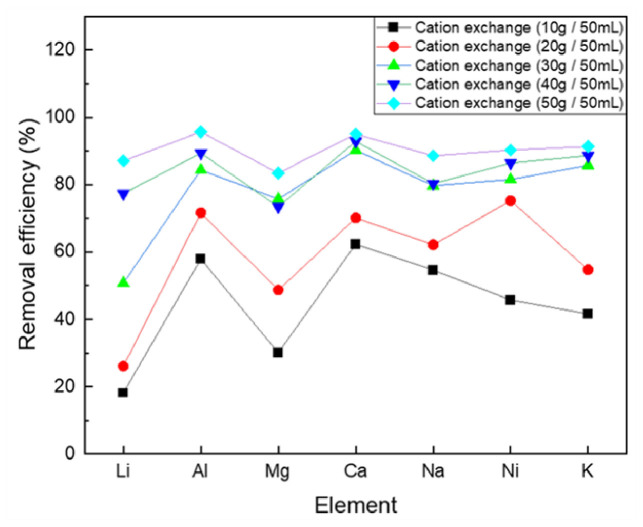
Removal efficiencies of ionic species as a function of cation-exchange resin dosage.

Figure 5 shows the removal efficiencies of the ionic species as a function of MC-10H dosage. The impurity-removal efficiency increased with increasing resin dosage, and at 50 g the removal efficiencies of Al, Mg, Ca, Na, Ni, and K reached 95.7%, 88.8%, 95.1%, 86.4%, 88.6%, and 91.3%, respectively. These results indicate that the –SO_3_H functional groups of MC-10H effectively removed dissolved cationic impurities through ion-exchange reactions.

**Figure 6:**
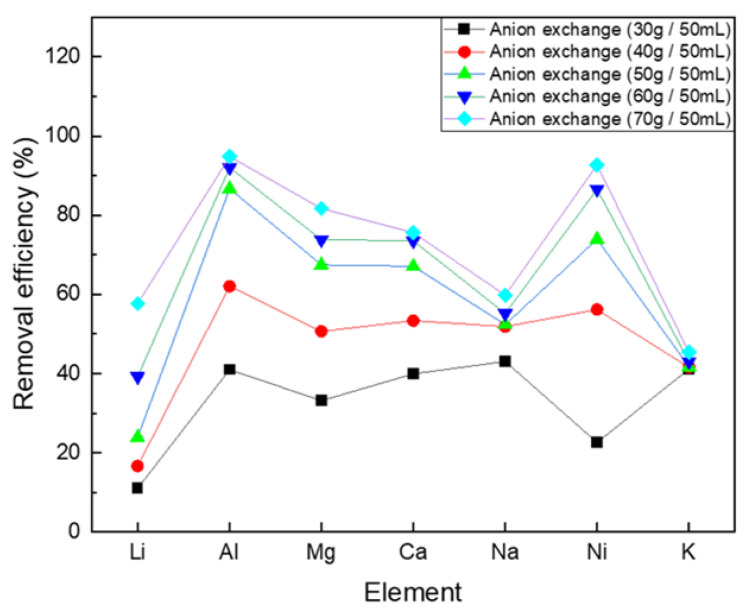
Removal efficiencies of ionic species as functions of anion-exchange resin dosage.

**Figure 7:**
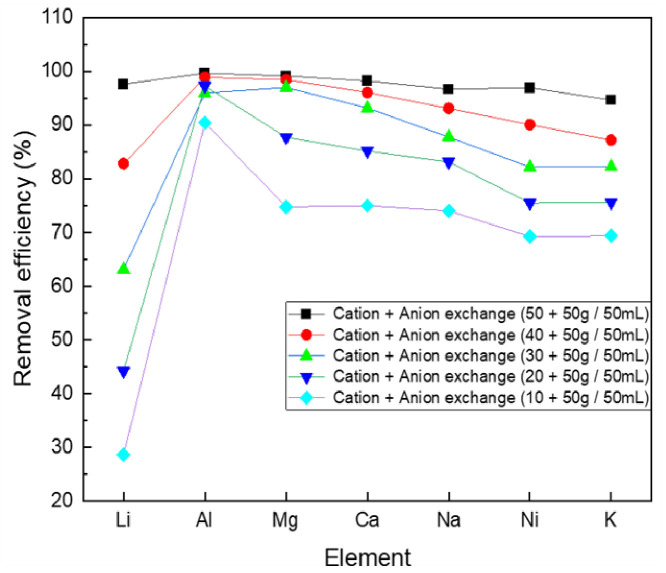
Removal efficiencies of ionic species as functions of cation-exchange resin dosage (anion-exchange resin dosage fixed at 50 g).

Because the ion-exchange experiments were conducted as single experimental runs for each resin-dosage condition, the removal efficiencies shown in Figure 5, Figure 6 and Figure 7 do not include standard deviations or error bars. Therefore, these results should be interpreted as trend-based process-screening data rather than statistically validated reproducibility data. Nevertheless, the systematic changes in impurity concentration with resin dosage provide useful information for identifying the appropriate pretreatment condition. Multivalent cations such as Al^3+^, Mg^2+^, Ca^2+^, and Ni^2+^ are generally expected to show relatively high affinity for the resin because of their higher charge. In the present study, however, when a sufficient resin dosage was provided, even monovalent ions such as Na^+^ and K^+^ exhibited removal efficiencies above 80%. At the highest dosage, all impurity cations showed similarly high removal efficiencies in the range of 85–96%. This behavior suggests that, when the number of available exchange sites becomes excessive relative to the ionic load, the charge-based selectivity of the resin becomes less pronounced and multiple cation species can be removed simultaneously to a similar extent. However, Li^+^ also showed a high removal efficiency of up to approximately 87%, despite its monovalent character and generally lower affinity for cation-exchange resins. This substantial lithium loss indicates that simply increasing the resin dosage is not a practical strategy for impurity removal in this system. In other words, a clear trade-off exists between impurity removal and lithium retention. Therefore, although cation-exchange treatment was effective for reducing metallic impurities, the resin dosage had to be carefully optimized to balance impurity removal against lithium loss. Overall, the cation-exchange process was confirmed to be effective as a pretreatment step for metallic impurity removal. At the same time, the significant co-removal of Li suggests that cation exchange alone is insufficient for selective purification of the Li_2_SO_4_ solution. These results support the need for subsequent anion-exchange treatment to further control the solution chemistry, particularly sulfate concentration and pH, while minimizing additional lithium loss.

#### 3.1.2. Anion-Exchange Treatment with MA-10OH Resin

The anion-exchange treatment was conducted using Trilite^®^ MA-10OH resin to evaluate both impurity removal behavior and pH adjustment in the Li_2_SO_4_ solution. The initial solution exhibited a strongly acidic condition (pH ≈ 1). The resin dosage was varied from 30 to 70 g, and all experiments were performed using 50 mL of the Li_2_SO_4_ leachate. The experimental conditions are summarized in Table 4.

Table 5 presents the concentration changes in ionic species as a function of MA-10OH dosage. As the resin dosage increased, the concentrations of most impurity elements, including Al, Mg, Ca, Na, Ni, and K, gradually decreased. In particular, at a dosage of 50 g, the concentrations of Al, Mg, and Ni were reduced to 535.0, 364.8, and 45.02 mg kg^−1^, respectively, indicating increased removal. A notable feature of the anion-exchange treatment was the substantial increase in solution pH with increasing resin dosage. At 30 and 40 g, the pH values were approximately 1–2 and 4, respectively. However, at 50 g, the pH increased sharply to 7–8, and further increased to 10–11 and 14 at 60 and 70 g, respectively. This pH increase can be attributed to the exchange of sulfate ions and protons with hydroxide ions released from the MA-10OH resin, resulting in the neutralization of acidity and accumulation of excess OH^−^ in the solution. It should be noted that the decrease in Al concentration observed at low MA-10OH dosage, particularly at pH 1–2, cannot be attributed primarily to Al hydroxide precipitation because Al is generally soluble under strongly acidic conditions. Therefore, the initial decrease in Al concentration at low pH is more likely related to non-precipitation pathways, such as interaction of Al-bearing sulfate complexes with the anion-exchange resin, adsorption or retention within the resin phase, solution hold-up in the resin beads, or physical entrapment during resin separation. As the resin dosage increased and the solution pH shifted toward near-neutral or alkaline conditions, pH-induced hydrolysis and possible precipitation of multivalent metal species may have contributed more significantly to impurity removal.

Figure 6 illustrates the removal efficiencies of ionic species as a function of MA-10OH dosage. The overall removal efficiency increased with increasing resin dosage. In particular, multivalent cations such as Al^3+^, Mg^2+^, Ca^2+^, and Ni^2+^ exhibited relatively high removal efficiencies, approximately 70–95%, at dosages of 50 g and above. However, the removal behavior should be interpreted in a pH-dependent manner. At low MA-10OH dosage, where the bulk solution pH remained strongly acidic, the decrease in Al concentration cannot be explained by hydroxide precipitation. Instead, it is more plausibly associated with the removal of Al-bearing sulfate complexes, resin-phase retention, adsorption, or physical entrapment. The higher removal efficiencies of Al, Mg, and Ni compared with those of Na and K at higher resin dosages are consistent with the stronger hydrolysis tendency and lower solubility of multivalent metal ions as the solution pH approaches near-neutral or alkaline conditions. Nevertheless, because no direct characterization of the precipitated or resin-deposited solids was performed, this interpretation should be regarded as an indirect explanation based on solution chemistry and concentration changes.

Although MA-10OH is fundamentally an anion-exchange resin, its OH-form functional groups exchange with anionic species such as SO_4_^2−^ and also contribute to neutralization of the initially acidic solution through the release of OH^−^. As a result, the solution pH increased markedly with increasing resin dosage. Therefore, impurity removal during MA-10OH treatment likely occurred through multiple pathways rather than through hydroxide precipitation alone. These pathways may include sulfate/proton exchange, removal of metal–sulfate complex species, resin-phase retention, physical entrapment, and, under near-neutral to alkaline conditions, pH-induced hydrolysis or precipitation of multivalent metal species. Because the solid products deposited on the resin surface or formed in the solution were not directly characterized by XRD, SEM-EDS, or precipitate composition analysis in this study, the formation of specific metal hydroxide phases cannot be conclusively confirmed. Therefore, the impurity-removal mechanism is interpreted here as a plausible combined pathway rather than as direct evidence of hydroxide precipitation.

In contrast, monovalent cations such as Na^+^ and K^+^ showed relatively limited removal efficiencies, approximately 40–60%, which can be attributed to their high solubility as hydroxides and lower tendency to form insoluble precipitates. This trend is consistent with general aqueous chemistry and ion-exchange behavior, where multivalent ions are preferentially removed under alkaline conditions. A gradual decrease in Li concentration was also observed with increasing resin dosage, particularly at pH values above 10. However, considering that Li^+^ does not readily participate in ion-exchange reactions with the MA-10OH resin and does not form insoluble hydroxide species, this decrease is likely not due to chemical removal. Instead, it is attributed to process-related factors such as physical entrapment on the resin surface, co-removal with fine precipitates during filtration, and minor losses during agitation. Such behavior is commonly observed as a process-related loss in ion-exchange pretreatment systems. Among the tested conditions, the near-neutral pH range, approximately 7–8, achieved at a resin dosage of 50 g provided a favorable balance between impurity removal and solution stability. This condition also preserves the intrinsic characteristics of the Li_2_SO_4_ solution while providing an optimal reaction environment for the subsequent Ba(OH)_2_ precipitation step. Therefore, the 50 g condition was selected as the optimal pretreatment condition for the anion-exchange process.

#### 3.1.3. Mixed Cation–Anion Exchange Treatment

Based on the results of the individual cation- and anion-exchange experiments, a mixed-resin treatment was designed to simultaneously improve impurity removal and solution conditioning. In particular, the anion-exchange resin was found to be effective for pH neutralization, whereas the cation-exchange resin showed high removal efficiencies for multivalent cations such as Al, Mg, and Ca. Therefore, simultaneous application of both resins was expected to maximize the pretreatment efficiency of the Li_2_SO_4_ leachate. In this section, a mixed ion-exchange process was applied using the cation-exchange resin Trilite^®^ MC-10H and the anion-exchange resin Trilite^®^ MA-10OH for impurity removal and optimization of the solution chemistry. The dosage of the cation-exchange resin was varied from 10 to 50 g, while the dosage of the anion-exchange resin was fixed at 50 g. The total volume of the feed solution was fixed at 50 mL. The experimental conditions are summarized in Table 6.

Table 7 shows the concentrations of ionic species and the pH values obtained after mixed cation–anion exchange treatment. As the dosage of the cation-exchange resin increased, the concentrations of major impurities, including Al, Mg, Ca, Na, Ni, and K, generally decreased. Under the 10 g cation/50 g anion condition, the concentrations of Al, Mg, Ca, Na, Ni, and K decreased to 384.2, 282.8, 79.10, 65.33, 61.33, and 53.14 mg kg^−1^, respectively, indicating substantial impurity removal. At the same condition, the Li concentration decreased from 2460 to 1756 mg kg^−1^, corresponding to a relatively moderate lithium loss compared with the higher cation-resin dosage conditions. As the dosage of the cation-exchange resin increased further, the impurity-removal efficiency improved, but the Li concentration decreased more sharply and the solution pH gradually declined. While the addition of 50 g of anion-exchange resin alone increased the pH from the initial strongly acidic condition to near-neutral values, increasing the cation-resin dosage caused the pH to decrease again. This trend is attributed to the release of H^+^ ions from the cation-exchange resin, which partially neutralized the OH^−^ ions introduced by the anion-exchange resin and thereby restored solution acidity to some extent. These results indicate that higher impurity-removal performance is accompanied by a greater burden on solution composition, and that the optimum condition cannot be determined solely on the basis of impurity-removal efficiency.

Figure 7 shows the removal efficiencies of ionic species as a function of cation-exchange resin dosage when the anion-exchange resin dosage was fixed at 50 g. The removal efficiencies of all impurity ions increased with increasing cation-resin dosage. In particular, Al showed a removal efficiency above 90% under all tested conditions. Mg and Ni also exhibited improved removal with increasing cation-resin dosage, reaching approximately 88–97% and 85–97%, respectively. Although monovalent cations such as Na^+^ and K^+^ showed lower removal efficiencies than multivalent ions, their removal also gradually improved with increasing resin dosage, reaching approximately 60–95%. Among the tested conditions, the combination of 10 g cation-exchange resin and 50 g anion-exchange resin provided the most balanced pretreatment performance. Under this condition, Al was removed by more than 90%, while Mg, Ca, Na, K, and Ni were removed at moderate but stable levels of approximately 60–75%. At the same time, lithium loss was still substantial at approximately 30%, which was the lowest among the mixed-resin conditions. In addition, the solution pH remained within the near-neutral range of 6–7, indicating that impurity removal, lithium retention, and pH stabilization were relatively balanced under this condition. Although increasing the resin dosage further improved impurity-removal efficiency, it also resulted in a significant increase in lithium loss. For example, under the 50 g cation/50 g anion condition, the removal efficiencies of most impurity ions exceeded 90%, but only approximately 2–5% of the initial Li concentration remained in solution. These results clearly demonstrate a trade-off between impurity removal, lithium retention, and pH control in the mixed ion-exchange system. Therefore, the condition employing 10 g of cation-exchange resin and 50 g of anion-exchange resin was selected as the optimal pretreatment condition for this study, considering the overall balance among impurity-removal efficiency, lithium retention, and pH stability. This condition was subsequently adopted for the following Ba(OH)_2_ double-displacement reaction.

### 3.2. LiOH Production by Precipitation with Ba(OH)_2_

#### 3.2.1. Reaction Behavior of LiOH Formation as a Function of Temperature and Molar Ratio

After impurity removal by the ion-exchange pretreatment described in Section 3.1, a purified Li_2_SO_4_ solution with stabilized pH was obtained. The final pretreated solution contained 1755 mg kg^−1^ Li and 17,500 mg kg^−1^ SO_4_^2−^. These concentrations correspond to approximately 0.253 M Li^+^, 0.126 M Li_2_SO_4_-equivalent concentration based on Li, and approximately 0.18 M SO_4_^2−^. The Ba(OH)_2_ dosage was determined based on the [OH]:[Li] molar ratio, using the measured Li concentration of the pretreated solution, rather than based solely on the measured sulfate concentration. Therefore, the 0.126 M value represents the Li_2_SO_4_-equivalent concentration calculated from Li, not the analytically measured SO_4_^2−^ concentration. LiOH was produced from 300 mL of the pretreated Li_2_SO_4_ solution by the addition of Ba(OH)_2_, and the effects of reaction temperature and [OH]:[Li] molar ratio on LiOH conversion behavior were investigated. The precipitation-conversion experiments were conducted by varying the [OH]:[Li] molar ratio to 1:1, 2:1, and 3:1, corresponding to Ba(OH)_2_ dosages of 6.50, 13.00, and 19.51 g, respectively. The reaction temperature was controlled at 60, 70, and 80 °C, resulting in a total of nine experimental conditions. The reaction was carried out in a Teflon beaker under an Ar atmosphere (100 mL min^−1^) for 2 h. After the reaction, the precipitated BaSO_4_(s) and the LiOH-containing filtrate were separated by vacuum filtration. The ionic concentrations in the filtrate were analyzed by ICP-OES and IC, and the solid product recovered after vacuum-Ar drying was characterized by XRD. The detailed experimental conditions are summarized in Table 8.

The filtrate compositions after the precipitation reaction are summarized in Table 9, which lists the measured Li and SO_4_^2−^ concentrations for each experimental condition. The residual SO_4_^2−^ concentration varied depending on both temperature and [OH]:[Li] molar ratio. Overall, the lowest residual sulfate concentration was observed under the 1:1 condition at each temperature. This result suggests that sulfate removal via BaSO_4_ precipitation proceeded most effectively when the hydroxide-to-lithium ratio was maintained at the stoichiometric level required for conversion. By contrast, when excess Ba(OH)_2_ was added under the 2:1 and 3:1 conditions, the residual SO_4_^2−^ concentration increased rather than decreased. This result indicates that sulfate removal in the present system was not governed solely by the thermodynamic driving force for BaSO_4_ formation, but was also strongly affected by precipitation kinetics, particle aggregation, and solid–liquid separation behavior. Under excess Ba(OH)_2_ conditions, the solution becomes highly alkaline and contains elevated concentrations of Ba^2+^ and OH^−^, resulting in increased ionic strength and rapid supersaturation. These conditions can promote instantaneous nucleation of fine or colloidal BaSO_4_ particles rather than the growth of larger and more readily filterable precipitates. As a result, some sulfate-containing fine particles or associated species may remain dispersed in the filtrate or pass through the filtration step, leading to an apparently higher residual sulfate concentration.

A comparison of the temperature effect further showed that the lowest residual SO_4_^2−^ concentration was obtained at 70 °C. This result suggests that, at 70 °C, the rates of precipitation formation, particle growth, and ionic diffusion were balanced most favorably for the Li_2_SO_4_–Ba(OH)_2_ system. At 80 °C, however, the residual sulfate concentration increased again under some conditions. This behavior may be associated with slight changes in the precipitation equilibrium at elevated temperature or with partial destabilization of the precipitation environment in the presence of excess ions. Therefore, the results indicate that 70 °C provided the most favorable condition for sulfate removal in the present system. The measured Li concentration in the filtrate also varied with reaction condition. In particular, the Li concentration increased from 1755 mg kg^−1^ in the pretreated feed solution to 2567 mg kg^−1^ in the filtrate under the 70 °C and [OH]:[Li] = 1:1 condition. This increase should not be interpreted as an increase in the total amount of lithium or as direct evidence of enhanced lithium conversion. Rather, it is most likely associated with a concentration effect caused by changes in liquid volume during the precipitation-conversion and filtration steps. Possible contributors include partial water evaporation during reaction at elevated temperature, solution hold-up in the BaSO_4_ precipitate or filter cake, retention of liquid in the filtration apparatus, and differences in recovered filtrate volume. Because the total liquid mass and volume before and after reaction were not quantitatively corrected in the present study, the absolute Li concentration in the filtrate was not used as the primary indicator for evaluating conversion behavior. Accordingly, in this section, the reaction tendency was mainly evaluated on the basis of the residual SO_4_^2−^ concentration in the filtrate, while condition-specific filtrate pH values and complete Li mass-balance data were not included because they were not systematically measured in the present study.

#### 3.2.2. Evaluation of Sulfate-Removal-Based Apparent Conversion Efficiency as a Function of Temperature and Molar Ratio

The progress of the Li_2_SO_4_-to-LiOH precipitation-conversion reaction was evaluated using a sulfate-removal-based apparent conversion efficiency. This value was calculated from the residual SO_4_^2−^ concentration in the filtrate after the reaction using Equation (5), where Q_0_ represents the initial amount of SO_4_^2−^ in the Li_2_SO_4_ solution and Q represents the amount of SO_4_^2−^ remaining in the filtrate after the conversion reaction. Because this calculation is based on sulfate removal through BaSO_4_ precipitation, it should be interpreted as an indirect index of the precipitation-conversion progress rather than as a direct lithium-based conversion efficiency to LiOH. Because the Li concentration in the filtrate may be affected by liquid-volume changes during reaction and filtration, including evaporation and filtrate recovery differences, the Li concentration was not used to calculate the conversion efficiency. Instead, the sulfate-removal-based apparent conversion efficiency was used as an indirect index of the precipitation-conversion progress. A complete Li mass balance will be required in future work to quantitatively determine lithium recovery efficiency.

(5) $$conversion\;\left(\%\right)=\left(1-\frac{Q}{Q_{0}}\right)\times 100$$

**Figure 8:**
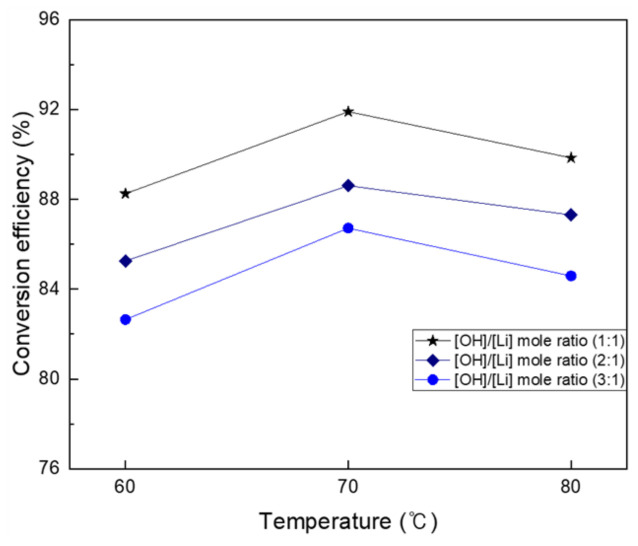
Variation in sulfate-removal-based apparent conversion efficiency with reaction temperature and [OH]:[Li] molar ratio.

The calculated sulfate-removal-based apparent conversion efficiencies are summarized in Table 10 and Figure 8. Table 10 provides a quantitative comparison of the apparent conversion efficiencies under different reaction temperatures and [OH]:[Li] molar ratios, whereas Figure 8 presents the same trend graphically for easier visualization of the temperature- and molar-ratio-dependent behavior. The apparent conversion efficiency calculated from the residual SO_4_^2−^ concentration in the filtrate showed a clear dependence on both reaction temperature and Ba(OH)_2_ dosage.

Under all tested temperatures, the 1:1 molar ratio yielded the highest sulfate-removal-based apparent conversion efficiency. In particular, the maximum value of 91.91% was obtained at 70 °C under the [OH]:[Li] = 1:1 condition. This result suggests that the reaction between Ba^2+^ and SO_4_^2−^ proceeded most effectively under the stoichiometric condition, enabling stable formation and separation of BaSO_4_ precipitates. When excess Ba(OH)_2_ was added under the 2:1 and 3:1 conditions, the sulfate-removal-based apparent conversion efficiency decreased despite the higher amount of Ba^2+^ supplied to the system. This behavior can be explained by the fact that excess Ba(OH)_2_ may create a high-pH and high-ionic-strength environment, leading to rapid BaSO_4_ nucleation and the formation of fine or colloidal precipitates. Such particles are less favorable for aggregation and filtration than well-grown BaSO_4_ crystals, and therefore a fraction of sulfate-containing species may remain in the filtrate after solid–liquid separation. Accordingly, the lower apparent conversion efficiency under the 2:1 and 3:1 conditions is attributed mainly to precipitation–separation behavior under excess reagent conditions rather than to insufficient thermodynamic driving force for BaSO_4_ formation. The effect of temperature was also clearly observed. At 60 °C, the conversion efficiency was lower than that at 70 °C, suggesting that ionic diffusion and precipitation growth were not sufficiently promoted at the lower temperature. By contrast, at 70 °C, the conversion efficiency reached its maximum value under all [OH]:[Li] conditions, indicating that this temperature provided the most favorable balance between ion transport and precipitation behavior in the present system. At 80 °C, the conversion efficiency decreased again, implying that the precipitation-conversion behavior was slightly destabilized at the elevated temperature. This temperature dependence can be qualitatively interpreted in terms of the precipitation–dissolution equilibrium of BaSO_4_, as expressed in Equation (6).

Ba^2+^ + SO_4_^2−^ ⇌ BaSO_4_(s) (6)

At an elevated temperature, slight changes in the precipitation equilibrium may increase the residual SO_4_^2−^ concentration in the filtrate, thereby lowering the apparent conversion efficiency. Although the conversion of Li_2_SO_4_ to LiOH is thermodynamically favorable over the investigated temperature range, the observed differences in conversion efficiency appear to be governed more strongly by precipitation kinetics and solid–liquid separation behavior than by the overall thermodynamic driving force alone. In addition, the pH of the filtrate remained above 12 after the reaction under all tested conditions, corresponding to the stability region of LiOH in the Eh–pH diagram. This result indicates that the reaction system was sufficiently alkaline for LiOH stability throughout the investigated temperature range. Although some variation in Li concentration was observed depending on the reaction condition, this variation is considered to be influenced by differences in solution recovery and minor experimental losses, and therefore it was not used as the primary indicator for evaluating the conversion behavior. Overall, as shown in Table 11 and Figure 8, the precipitation-conversion process using Ba(OH)_2_ achieved the highest conversion efficiency of 91.91% at 70 °C and [OH]:[Li] = 1:1. This condition was therefore considered the optimum for LiOH conversion in the present study.

**Figure 9:**
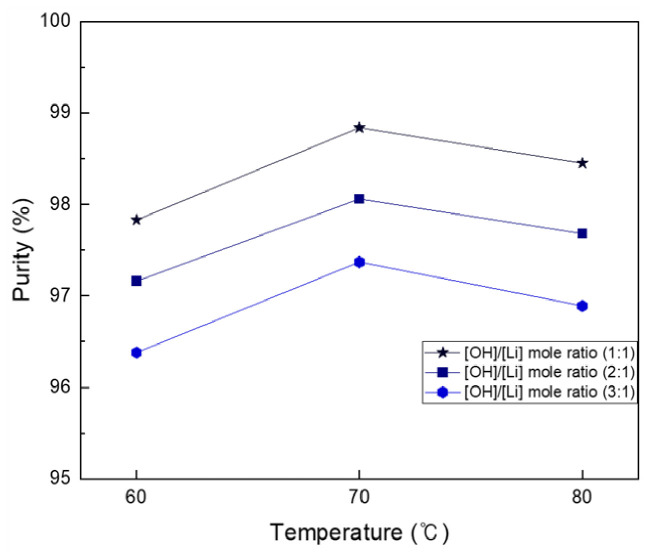
Purity of LiOH in solution as a function of reaction temperature and [OH]:[Li] molar ratio.

It should be noted that the precipitation-conversion experiments were also conducted as single experimental runs for each temperature and [OH]:[Li] molar-ratio condition. Accordingly, the conversion efficiencies and LiOH purities shown in Figure 8 and Figure 9 are presented without standard deviations or error bars. These data are intended to compare the relative effects of reaction temperature and reagent dosage and to identify a preliminary optimum condition. Further replicate experiments are required to quantitatively evaluate experimental variability and confirm reproducibility.

#### 3.2.3. Evaluation of the Purity of LiOH Solution as a Function of Temperature and Molar Ratio

The purity of the LiOH solution was evaluated from the filtrate composition after the reaction. The concentrations of Li^+^ and major impurity ions, including Ba^2+^, Mg^2+^, Ca^2+^, Al^3+^, Na^+^, and K^+^, were determined by ICP-OES analysis and converted to a LiOH basis for purity estimation. The purity results are summarized in Table 11 and Figure 9. Table 11 provides the calculated purity values under different reaction temperatures and [OH]:[Li] molar ratios, whereas Figure 9 presents the same trends graphically.

As with the conversion efficiency, the filtrate-based purity showed a clear dependence on the reaction conditions. However, the controlling factors for purity differed somewhat from those governing sulfate removal. In particular, the purity was more strongly influenced by the solubility of impurity ions, the extent of precipitation of impurity-bearing phases, and the speciation behavior of Li^+^ and OH^−^ in solution. At 60 °C, the purity was lower than that observed at higher temperatures. This behavior suggests that, at the lower temperature, ionic diffusion and precipitation kinetics were not sufficiently promoted, allowing not only residual sulfate but also more of impurity ions such as Mg^2+^, Ca^2+^, and Al^3+^ to remain in the filtrate. As a result, the calculated LiOH purity was relatively low under this condition. At 70 °C, both the reaction and precipitation processes proceeded more favorably, resulting in the highest filtrate purity. Under this condition, BaSO_4_ precipitation and the co-removal of some impurity ions appear to have been most effective, thereby minimizing the concentration of dissolved impurities in the filtrate. In particular, the highest purity of 98.84% was obtained at 70 °C under the [OH]:[Li] = 1:1 condition. This result indicates that the stoichiometric condition provided the most favorable balance between sulfate removal, impurity suppression, and LiOH stabilization in solution.

When the [OH]:[Li] molar ratio was increased to 2:1 and 3:1 at the same temperature, the purity decreased slightly. This trend suggests that excess OH^−^ and Ba^2+^ may modify the ionic environment of the solution and influence the dissolution or stabilization behavior of impurity species. Under highly alkaline conditions, some impurity ions may remain in solution as soluble hydroxide or complex species, thereby lowering the apparent purity of the LiOH-containing filtrate. At 80 °C, the purity remained relatively high but was slightly lower than that at 70 °C. Although the overall precipitation reaction remained favorable, the elevated temperature may have altered the precipitation–dissolution balance and increased the residual concentration of certain impurity ions in the filtrate. Therefore, while the conversion still proceeded efficiently at 80 °C, the resulting filtrate purity was somewhat lower than the maximum value observed at 70 °C. Overall, the LiOH purity evaluated on the basis of the filtrate composition followed the order of 60 °C < 80 °C < 70 °C. In addition, the [OH]:[Li] = 1:1 condition consistently yielded the highest purity at all investigated temperatures, whereas excess hydroxide addition resulted in slightly lower purities. Therefore, from the viewpoint of solution composition, 70 °C and [OH]:[Li] = 1:1 were considered the most appropriate operating conditions for suppressing metallic impurities in the LiOH-containing filtrate.

It should be emphasized that the purity value reported in Table 11 was calculated from the filtrate composition mainly on the basis of Li and metallic impurity concentrations. Therefore, this value represents a filtrate-based LiOH-equivalent purity with respect to metallic impurities, rather than the sulfate-inclusive mass purity of the final dried solid product. Under the optimal condition of 70 °C and [OH]:[Li] = 1:1, the filtrate still contained 1414 mg kg^−1^ SO_4_^2−^. If this residual sulfate remains in the solution during evaporation and drying, it may be incorporated into the recovered solid as residual Li_2_SO_4_ or other sulfate-containing salts, thereby lowering the actual mass purity of the dried product. For example, assuming that all residual SO_4_^2−^ remains as Li_2_SO_4_ after drying, part of the dissolved Li^+^ would be associated with sulfate rather than LiOH. In this case, the sulfate-inclusive solid purity would be substantially lower than the filtrate-based LiOH-equivalent purity. Therefore, the value of 98.84% should not be interpreted as the final dried-solid purity. Instead, it should be regarded as an indicator of the degree of metallic impurity removal in the LiOH-containing filtrate. Accurate determination of the final product purity requires direct chemical analysis of the dried solid and a complete mass balance including Li, SO_4_^2−^, Ba, residual metallic impurities, and water loss during crystallization and drying.

To further clarify the origin of the remaining impurity burden, the full impurity profile of the final LiOH-containing filtrate obtained under the optimal condition of 70 °C and [OH]:[Li] = 1:1 is summarized in Table 12. Under this condition, the filtrate contained 2567 mg kg^−1^ Li and 1414 mg kg^−1^ residual SO_4_^2−^. The presence of residual sulfate indicates that BaSO_4_ precipitation was highly effective but not completely exhaustive. Therefore, residual sulfate species still represent one of the important factors limiting the final LiOH purity. In addition to sulfate, residual Ba originating from the added Ba(OH)_2_ and soluble alkali or alkaline-earth impurities such as Na, K, Mg, and Ca are considered the main contributors to the impurity fraction remaining in the filtrate. Among these elements, Na and K are difficult to remove by hydroxide precipitation because of their high solubility under alkaline aqueous conditions. Mg and Ca can be partially removed through hydroxide or sulfate-related precipitation, but their complete removal is limited by solution speciation, ionic strength, and possible re-dissolution under highly alkaline conditions. Al and Ni were effectively reduced during the ion-exchange pretreatment; however, trace amounts may persist depending on hydrolysis behavior and solid–liquid separation efficiency. Residual Ba may also remain in the filtrate when the precipitation and separation of BaSO_4_ are not completely quantitative. These residual species explain why the maximum LiOH purity reached 98.84% but remained below the typical battery-grade LiOH·H_2_O target of >99.5%. It should also be noted that the purity in this study was evaluated based on the filtrate composition before drying. During subsequent evaporation, crystallization, and drying, soluble impurities remaining in the mother liquor may become enriched as water is removed. These impurities may be incorporated into the recovered solid product through mother-liquor entrainment, adsorption on crystal surfaces, or co-crystallization of minor residual salts. Therefore, further deep purification steps are required to prevent impurity enrichment during crystallization and drying. Possible approaches include secondary polishing ion exchange, additional sulfate removal, fine filtration before crystallization, washing of LiOH·H_2_O crystals, and recrystallization under CO_2_-free and impurity-controlled conditions. A limitation of the present study is that the experimental data were obtained from single runs without replicate measurements. Therefore, the absence of error bars in Figure 5, Figure 6, Figure 7, Figure 8 and Figure 9 limits the statistical assessment of reproducibility. Future work should include repeated experiments under the selected optimum conditions, preferably in triplicate, to determine standard deviations and confidence intervals for impurity removal efficiency, LiOH conversion efficiency, and product purity. Such validation will be essential for robust process optimization and scale-up.

#### 3.2.4. Crystallization and Phase Identification of the Final Products

**Figure 10:**
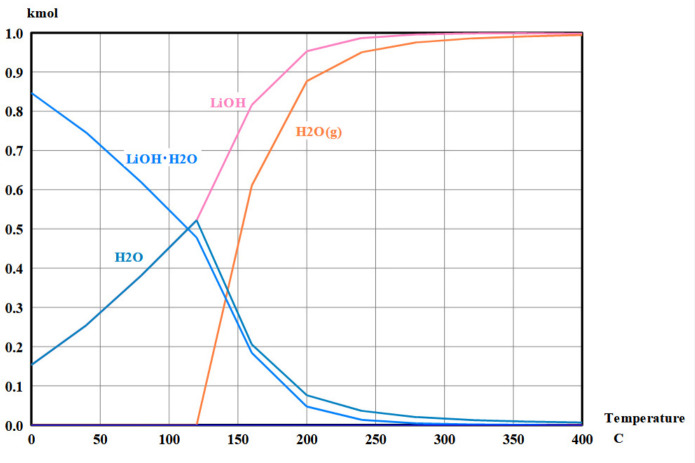
Calculated phase fractions of LiOH·H_2_O, LiOH, and Li_2_O as a function of temperature using HSC Chemistry 6.

The drying temperature for recovery of the LiOH product from the post-reaction filtrate was determined on the basis of thermodynamic calculations performed using HSC Chemistry 6. Figure 10 shows the calculated phase fractions of LiOH·H_2_O, LiOH, and Li_2_O as a function of temperature. According to the calculation, LiOH·H_2_O was the dominant phase below approximately 100 °C, whereas anhydrous LiOH appeared only partially in this temperature range. Based on this result, 90 °C was selected as the drying temperature to enable solid recovery while maintaining the stability of the monohydrate phase.

**Figure 11:**
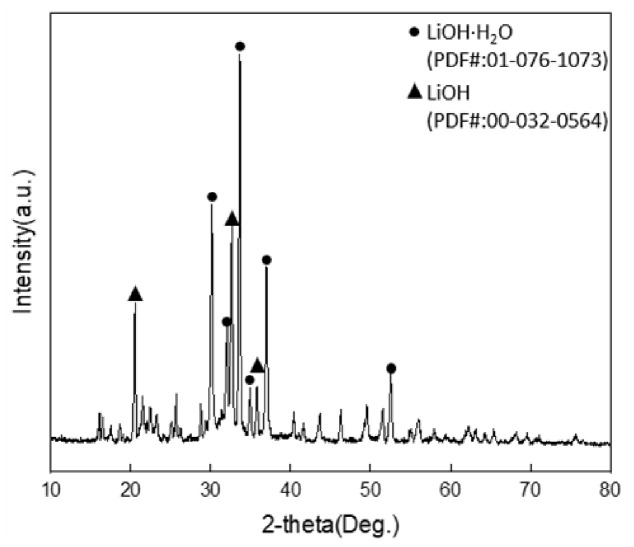
XRD pattern of the solid product obtained after drying at 90 °C under a vacuum-Ar atmosphere.

The LiOH-containing filtrate obtained after the precipitation-conversion reaction was dried at 90 °C for 24 h under a vacuum-Ar atmosphere to recover the solid product. The phase composition of the dried powder was analyzed by X-ray diffraction (XRD), and the result is presented in Figure 11. Characteristic diffraction peaks corresponding to LiOH·H_2_O (PDF#01-076-1073) were clearly observed, together with several peaks assignable to anhydrous LiOH (PDF#00-032-0564). This result indicates that partial dehydration occurred during drying at 90 °C, while the monohydrate phase remained as the dominant crystalline product, consistent with the thermodynamic prediction shown in Figure 10. No significant amorphous features were observed, suggesting that the crystallinity of the product was preserved during the drying process.

In this study, the recovered solid product was primarily characterized by XRD to confirm the crystalline phases of LiOH·H_2_O and LiOH. Morphological characterization and particle-size analysis were not included because the main focus of this work was the solution-based purification and precipitation-conversion behavior rather than optimization of the crystallization morphology. However, for practical use of the recovered LiOH-containing product as a cathode precursor source, further characterization will be necessary. In particular, SEM/EDS analysis should be performed to examine the particle morphology and elemental distribution, while particle-size distribution analysis should be carried out to evaluate crystallization behavior and handling properties. Direct chemical analysis of the dried solid product will also be required to determine the sulfate-inclusive product purity and compositional uniformity.

**Figure 12:**
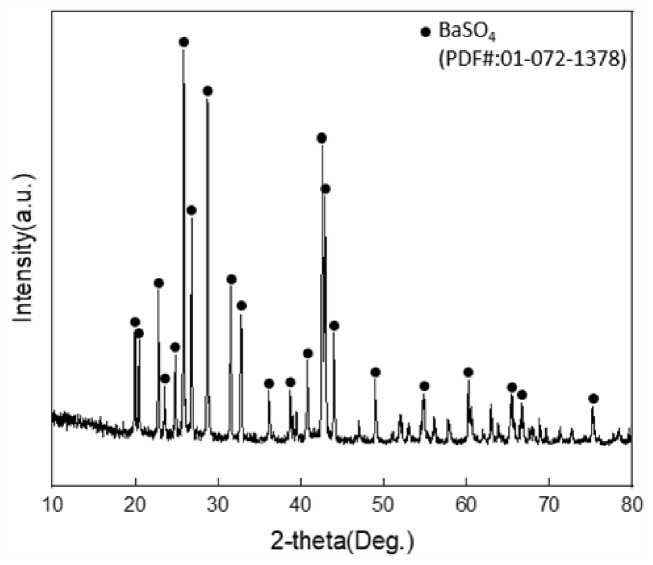
XRD pattern of the BaSO_4_ precipitate dried at 100 °C.

The evaluation of conversion efficiency and purity in this study was performed using the filtrate rather than the dried solid product. Because LiOH solids are susceptible to compositional variation caused by moisture uptake and surface reactions during drying and storage, quantitative impurity analysis was considered more reliable in the solution state. Accordingly, the recovered solid product was primarily used for phase identification by XRD, whereas the quantitative assessment of conversion and purity was based on the filtrate composition. The BaSO_4_ precipitate generated during the conversion reaction was analyzed separately to confirm the completeness of sulfate removal and solid-phase separation. The precipitate was dried at 100 °C for 24 h and then characterized by XRD. As shown in Figure 12, the diffraction peaks were in good agreement with those of BaSO_4_ (PDF#01-072-1378), and no additional crystalline phases or significant amorphous features were detected. This result confirms that the precipitate formed during the conversion process consisted predominantly of single-phase BaSO_4_.

Overall, the solid product recovered at 90 °C consisted of both LiOH·H_2_O and LiOH phases, showing good qualitative agreement between the thermodynamic phase prediction and the experimentally observed phase assemblage. In addition, the sulfate ions removed during the conversion process were successfully immobilized as crystalline BaSO_4_, and the XRD result confirmed that solid-phase separation proceeded effectively under the present experimental conditions.

It is important to distinguish the present study from previous work on BaSO_4_-driven LiOH·H_2_O recovery from spent lithium-containing sagger crucibles. The previous study mainly demonstrated the feasibility of converting lithium-bearing sagger-derived solution into LiOH·H_2_O using Ba(OH)_2_-based double decomposition. In contrast, the present work focuses on the pretreatment and purification of impurity-rich Li_2_SO_4_ leachate before the conversion step. The systematic comparison of cation-exchange, anion-exchange, and mixed-resin treatment provides information on impurity-removal behavior, lithium retention, and pH control, which were not the central focus of the previous study. In addition, the present study identifies residual sulfate and soluble metallic impurities as key limitations for achieving true battery-grade LiOH·H_2_O purity after crystallization. These findings provide practical guidance for designing a more complete purification–conversion process rather than only demonstrating the feasibility of the BaSO_4_ precipitation reaction. A detailed comparison between the previous BaSO_4_-driven LiOH recovery study and the present study is summarized in Table 13.

## 4. Conclusions

This study investigated a stepwise process for impurity control and lithium hydroxide production from lithium sulfate (Li_2_SO_4_) leachate generated from spent sagger crucibles used in the production of cathodes for LIBs by sulfuric acid leaching, with the aim of identifying suitable operating conditions for high-purity LiOH production. The initial leachate contained high concentrations of impurities, including Mg, Ca, Al, Na, and K, making it unsuitable for direct LiOH production. Therefore, a pretreatment process using cation- and anion-exchange resins was applied. This pretreatment effectively reduced the impurity levels in the solution and demonstrated its potential applicability to larger-scale process operation. Subsequent conversion of Li_2_SO_4_ using Ba(OH)_2_ showed that the reaction temperature and the [OH]:[Li] molar ratio had significant effects on both the sulfate-removal-based apparent conversion efficiency and product purity. In particular, under the condition of 70 °C and [OH]:[Li] = 1:1, sulfate ions were effectively removed through the precipitation of stable solid-phase BaSO_4_, while some metallic impurities were simultaneously reduced through combined effects such as resin-assisted removal, decreased solubility, and possible hydrolysis or precipitation under near-neutral to alkaline conditions. Under these conditions, the solution composition was most favorably stabilized, resulting in the highest sulfate-removal-based apparent conversion efficiency of 91.9% and the highest filtrate-based LiOH purity of 98.8%. In contrast, under excess OH^−^ conditions, the residual concentration of impurity species increased, indicating that over-alkaline conditions were unfavorable for obtaining high-purity LiOH. The post-reaction filtrate was dried at 90 °C under a vacuum-Ar atmosphere, and XRD analysis confirmed that the recovered solid consisted of coexisting LiOH·H_2_O and LiOH phases. Separate XRD analysis of the precipitate confirmed the formation of single-phase BaSO_4_, indicating that sulfate ions present in the leachate were stably separated into the solid phase during the conversion process. Because BaSO_4_ possesses very low solubility and high chemical stability, the precipitate has potential for further industrial utilization rather than simple disposal.

Further study is still needed to improve the practical applicability of the proposed process. In particular, direct characterization of the solids formed during the ion-exchange pretreatment should be performed in future work. XRD, SEM-EDS, and chemical analysis of the precipitates or resin-deposited solids would help distinguish the relative contributions of ion exchange, pH-induced precipitation, and physical entrapment to the removal of each impurity element. In addition, because the LiOH purity obtained in this study is still below the typical industrial target for battery-grade LiOH·H_2_O (>99.5%), the residual impurity profile of the final filtrate indicates that further deep purification is required. In particular, residual sulfate, Ba, and soluble alkali or alkaline-earth impurities such as Na, K, Mg, and Ca are considered critical factors limiting the final purity. These impurities may become further concentrated during evaporation, crystallization, and drying; therefore, secondary polishing purification, improved BaSO_4_ separation, crystal washing, and recrystallization should be considered in future work. In addition, complete mass-balance evaluation, including Li recovery, final filtrate volume, evaporative water loss, and liquid retained in the precipitate/filter cake, will be necessary to improve the quantitative precision of the process evaluation. Furthermore, because the present results were obtained from single experimental runs, repeated experiments with statistical evaluation will be required to verify reproducibility and establish reliable error ranges for impurity removal efficiency, sulfate-removal-based apparent conversion efficiency, and LiOH purity. Further characterization of the recovered LiOH-containing solid, including SEM/EDS analysis, particle-size distribution measurement, and direct chemical composition analysis, will also be necessary to evaluate morphology, compositional uniformity, and suitability as a lithium precursor for Li-ion battery cathode material synthesis.

Compared with previous work that mainly demonstrated BaSO_4_-driven LiOH·H_2_O recovery, the present study provides additional insight into the impurity-control stage required for processing spent-crucible-derived Li_2_SO_4_ leachate. In particular, the combined ion-exchange pretreatment results clarify the balance among impurity removal, Li retention, pH control, residual sulfate removal, and product-purity limitations, which are essential for developing a practical lithium recycling process. Overall, this study provides a process basis for the production of high-purity lithium hydroxide from Li_2_SO_4_ leachate recovered from spent reaction vessels and experimentally verifies the interconnection among impurity control, precipitation conversion, and crystallization. These results demonstrate the recycling potential of spent reaction vessels as a secondary lithium resource and are expected to contribute to improving the efficiency and economic feasibility of future industrial lithium recovery processes. With further optimization of lithium retention, product purity, process reproducibility, morphology and particle-size control, and scale-up, the proposed route is expected to provide a practical basis for lithium recycling applications.

## Figures and Tables

**Table 1:** Chemical Composition of Li_2_SO_4_ Leachate Obtained from Spent Reaction Vessels.

Element	mg/kg	Element	mg/kg	Element	mg/kg
Ag	<0.001	Cr	1.43	P	<17.28
Al	4021	Cu	<0.001	Pb	<0.001
As	<0.001	Fe	45.25	Sb	<0.001
B	4.81	K	173.6	Se	<0.001
Ba	<0.001	Li	2460	Sr	2.09
Be	<0.001	Mg	1122	Ti	20.41
Bi	1.07	Mo	<0.001	Tl	<0.001
Ca	316.7	Mn	19.7	V	<0.001
Cd	<0.001	Na	251.6	Zn	1.15
Co	28.79	Ni	172.8	SO_4_^2−^	100,000

**Table 2:** Experimental conditions for cation-exchange treatment with MC-10H resin.

Parameter	Condition
Variable condition	Resin dosage: 10, 20, 30, 40, and 50 g
Equipment	Magnetic stirrer
Stirring speed	200 rpm
Stirring time	1 h
Washing solution	Distilled water
Washing volume	500 mL
Sample volume	50 mL

**Table 3:** Concentration changes in ionic species in Li_2_SO_4_ solution after cation-exchange treatment with MC-10H resin.

Resin Dosage (g/50 mL)	Li	Al	Mg	Ca	Na	Ni	K	pH
Initial	2460	4021	1122	316.7	251.6	172.8	173.6	1
10	2013	1691	778.0	119.2	114.2	93.72	101.4	1
20	1818	1139	575.1	94.65	92.04	70.93	78.62	1
30	1013	629.4	272.0	90.95	51.17	35.93	24.75	1
40	557.9	426.1	202.0	22.49	42.23	27.10	19.69	1
50	317.7	174.1	125.9	15.81	34.20	19.64	15.04	1

**Table 4:** Experimental conditions for anion-exchange treatment with MA-10OH resin.

Parameter	Condition
Variable condition	Resin dosage: 30, 40, 50, 60, and 70 g
Equipment	Magnetic stirrer
Stirring speed	200 rpm
Stirring time	1 h
Washing solution	Distilled water
Washing volume	500 mL
Sample volume	50 mL

**Table 5:** Concentration changes in ionic species in Li_2_SO_4_ solution after anion-exchange treatment with MA-10OH resin.

Resin Dosage (g/50 mL)	Li	Al	Mg	Ca	Na	Ni	K	pH
Initial	2460	4021	1122	316.7	251.6	172.8	173.6	1
30	2188	2371	1213	190.1	142.7	133.7	102.2	1–2
40	2050	1524	749.6	147.7	131.2	75.75	101.2	4
50	1871	535.0	364.8	83.78	119.9	45.02	98.93	7–8
60	1494	320.4	293.8	35.60	112.9	23.23	95.50	10–11
70	1380	285.2	270.3	28.41	110.3	21.57	93.12	14

**Table 6:** Experimental conditions for ion-exchange treatment with mixed MC-10H and MA-10OH resins.

Parameter	Condition
Variable condition	Cation-exchange resin dosage: 10, 20, 30, 40, and 50 g
Equipment	Magnetic stirrer
Stirring speed	200 rpm
Stirring time	1 h
Water solution	Distilled water
Washing volume	500 mL
Sample volume	50 mL
Fixed condition	Anion-exchange resin dosage: 50 g

**Table 7:** Concentrations of ionic species and pH values after mixed cation–anion exchange treatment using MC-10H and MA-10OH resins.

Cation Resin (g)/Anion Resin (g)	Li	Al	Mg	Ca	Na	Ni	K	pH
Initial	2460	4021	1122	316.7	251.6	172.8	173.6	1
10/50	1755	384.2	282.8	79.10	65.33	61.33	53.14	6–7
20/50	1373	109.3	151.5	60.55	50.60	46.92	42.37	4–5
30/50	906.4	62.12	61.69	42.64	37.76	33.48	30.80	3–4
40/50	422.5	43.90	46.11	22.14	17.34	17.13	22.23	1
50/50	57.56	12.30	39.16	5.62	8.33	5.23	9.23	1

**Table 8:** Experimental conditions for LiOH precipitation using Ba(OH)_2_.

Parameter	Condition
Variable condition	Ba(OH)_2_ dosage: 6.50, 13.00, and 19.51 g
	Reaction temperature: 60, 70, and 80 °C
Fixed condition	Li_2_SO_4_ solution: 300 mL (0.126 M)
	Li concentration: 1755.48 mg kg^−1^
	SO_4_^2−^ concentration: 17,500 mg kg^−1^
	Gas supply: Ar (100 mL min^−1^)
	Stirring speed: 200 rpm
	Reaction time: 2 h

**Table 9:** Li and SO_4_^2−^ concentrations in the filtrate after Ba(OH)_2_ precipitation under different temperatures and molar ratios.

[OH]:[Li]	Temp (°C)	Li (mg kg^−1^)	SO_4_^2−^ (mg kg^−1^)
1:1	60	1891	2056
2:1	60	1814	2579
3:1	60	1767	3041
1:1	70	2567	1414
2:1	70	2253	1996
3:1	70	2161	2324
1:1	80	2013	1771
2:1	80	1972	2218
3:1	80	1959	2699

**Table 10:** Sulfate-removal-based apparent conversion efficiencies under different reaction temperatures and [OH]:[Li] molar ratios.

Temp (°C)	[OH]:[Li] = 1:1 (%)	[OH]:[Li] = 2:1 (%)	[OH]:[Li] = 3:1 (%)
60	88.3	85.3	82.7
70	91.9	88.6	86.7
80	89.9	87.3	84.6

**Table 11:** Purity of LiOH in the filtrate as a function of reaction temperature and [OH]:[Li] molar ratio.

Temp (°C)	[OH]:[Li] = 1:1 (%)	[OH]:[Li] = 2:1 (%)	[OH]:[Li] = 3:1 (%)
60	97.8	97.2	96.4
70	98.8	98.1	97.4
80	98.5	97.7	96.9

**Table 12:** Full impurity profile of the final LiOH-containing filtrate obtained under the optimal condition of 70 °C and [OH]:[Li] = 1:1.

Species	Concentration in Filtrate (%)	Possible Origin	Relevance to LiOH Purity
Ba	0.14	Residual Ba from Ba(OH)_2_ addition	Possible soluble Ba carryover
Na	0.31	Residual alkali impurity from leachate	Highly soluble; difficult to remove by precipitation
Mg	0.25	Incomplete removal during pretreatment	May persist as soluble or fine colloidal species
Al	0.46	Residual amphoteric impurity	May remain under specific pH conditions

**Table 13:** Comparison between the previous BaSO_4_-driven LiOH recovery study and the present work.

Item	Previous Study	Present Study
Primary objective	Demonstration of LiOH·H_2_O recovery via BaSO_4_-driven double decomposition	Development of an integrated purification–conversion route for impurity-rich Li_2_SO_4_ leachate
Feed characteristics	Lithium-containing spent sagger-derived material or solution	Spent-crucible-derived Li_2_SO_4_ leachate containing Na, K, Mg, Ca, Al, Ni, and high SO_4_^2−^
Main process emphasis	Ba(OH)_2_-mediated conversion and LiOH·H_2_O crystallization	Ion-exchange pretreatment, impurity control, lithium retention, and subsequent Ba(OH)_2_ conversion
Pretreatment investigation	Not the central focus	Systematic evaluation of cation-exchange, anion-exchange, and mixed-resin treatment
Key operating variables	Conversion and crystallization conditions	Resin dosage, pH control, Li loss, [OH]:[Li] molar ratio, and reaction temperature
Impurity-control analysis	Mainly product recovery-oriented	Analysis of impurity-removal behavior, residual sulfate, and soluble impurity limitations
Main contribution	Feasibility of LiOH·H_2_O recovery from spent sagger-derived lithium source	Process-level understanding of purification bottlenecks and requirements for practical LiOH recovery
Limitations clarified	Conversion and crystallization feasibility	Residual sulfate, cationic impurities, Li mass balance, reproducibility, and true dried-solid purity

## Data Availability

The original contributions presented in this study are included in the article. Further inquiries can be directed to the corresponding author.
